# Osteonecrosis of the jaw in a patient under treatment of osteoporosis with oral bisphosphonate

**DOI:** 10.4322/acr.2020.186

**Published:** 2020-12-14

**Authors:** Marília A. Figueiredo, Frederico Buhatem Medeiros, Karem López Ortega

**Affiliations:** 1 Universidade de São Paulo (USP), Faculdade de Odontologia, Departamento de Estomatologia, Centro de Atendimento a Pacientes Especiais, São Paulo, SP, Brasil

**Keywords:** Bisphosphonate-Related Osteonecrosis of the Jaw, Ibandronic Acid, Jaw, Osteonecrosis

## Abstract

Although uncommon in patients under oral therapy, bisphosphonate-related osteonecrosis of the jaw (BRONJ) can be a very severe issue. Early intervention with surgical resection should be the preferable method of treating any stage of the disease, resulting in better outcomes and decreasing the morbidity of this condition. A 77-year-old female patient attended the Special Care Dentistry Centre of the University of São Paulo Faculty of Dentistry (CAPE FOUSP) complaining mainly of “an exposed bone that appeared after tooth extraction performed six months earlier”. The patient was diagnosed with osteonecrosis associated with bisphosphonate (sodium ibandronate) and surgically treated with removal of bone sequestration and antibiotic therapy. The patient was followed up for six years (a total of 6 appointments), presenting good general health and no sign of bone exposure. Imaging findings showed no changes related to BRONJ either.

## INTRODUCTION

Osteoporosis is a bone metabolic disorder (BMD) characterized by loss of bone mineral density. Its treatment aims to prevent fractures by means of administration of medications, such as bisphosphonates (BPs).^1^

BPs bind to hydroxyapatite crystals, internalizing into the bone matrix and can be absorbed by osteoclasts during bone resorption (leading to apoptosis), excreted or re-captured, and re-incorporated into the skeleton. This characteristic results in a lengthy, continuous interference with bone turnover and whose bone retention time is estimated to be between 1 and 10 years.^2^ BPs can be classified depending on their action pattern, that is, nitrogenated or non-nitrogenated, with nitrogen-containing BPs (e.g., pamidronate, alendronate, risedronate, ibandronate, and zoledronate) being mostly used because they are more potent ^1^. Their absorption depends on the gender, age and bone resorption rate of the patient at the time, in addition to the administration route, since absorption of BPs is less than 1% when orally administered compared to the endogenous route, which can reach 50%.^2^ ^,^ ^3^

Inhibition of osteoclastic activity, in association with anti-anti-angiogenic properties, results consequently in decreased bone turnover and hyper-mineralization as well as accounts for bone hypo-vascularisation. These characteristics added to the inhibition of T cells’ function, macrophages and monocytes would lead to osteonecrosis of the jaw - one of the most significant complications of BPs.^2^ ^,^ ^3^

Several risk factors are associated with the development of bisphosphonate-related osteonecrosis of the jaw (BRONJ), such as invasive dental procedures (e.g., tooth extraction), inflammatory and/or infectious conditions, advanced age and dosage and length of treatment with BPs. Drug route is also closely related to the incidence of BRONJ, which occurs more frequently when administered intravenously (1.6 to 14.8%) than orally (0.001% to 0.01%).^4^ ^-^ ^7^

Although oral route accounts for a few cases of BRONJ, they may occur and present significant morbidity to the patient,^8^ leading to pathological fractures of the jaw.^9^ Therefore, it is important to recognize the clinical characteristics and the risk factors related to the development of BRONJ in these patients.^10^

## CASE REPORT

A 77-year-old female patient attended the Special Care Dentistry Centre of the University of São Paulo Faculty of Dentistry (CAPE FOUSP) complaining mainly of “an exposed bone that appeared after tooth extraction performed six months earlier”.

During medical history taking, the patient reported no history of chronic use of steroids or radiotherapy in the head and neck region. However, she reported painful swelling in the mentonian region, including abscess and fistula, after 1 year of dental treatment. In this clinical context, the dentist performed periodontal therapy by scraping all the teeth and extracting teeth 33 and 34, including antibiotic therapy (cephalexin and amoxicillin with potassium clavulanate). However, bone exposure advanced.

The patient was on medications for control of type-2 diabetes mellitus (glargine insulin and sitagliptin phosphate), cardiopathy, and systemic hypertension (salicylic acetyl acid, atenolol, enalapril maleate, and hydrochlorothiazide) and dyslipidemia (simvastatin and ciprofibrate). She also reported undergoing cardiac surgeries (2 mammary and 1 saphenous bypass grafts) in 2005 and thyroidectomy in 2011 (using levothyroxine sodium). The patient was also taking cholecalciferol and tribasic calcium phosphate for control of osteoporosis/osteopenia for many years as well as sodium ibandronate for two years. Pantoprazole was also used.

**Figure 1:**
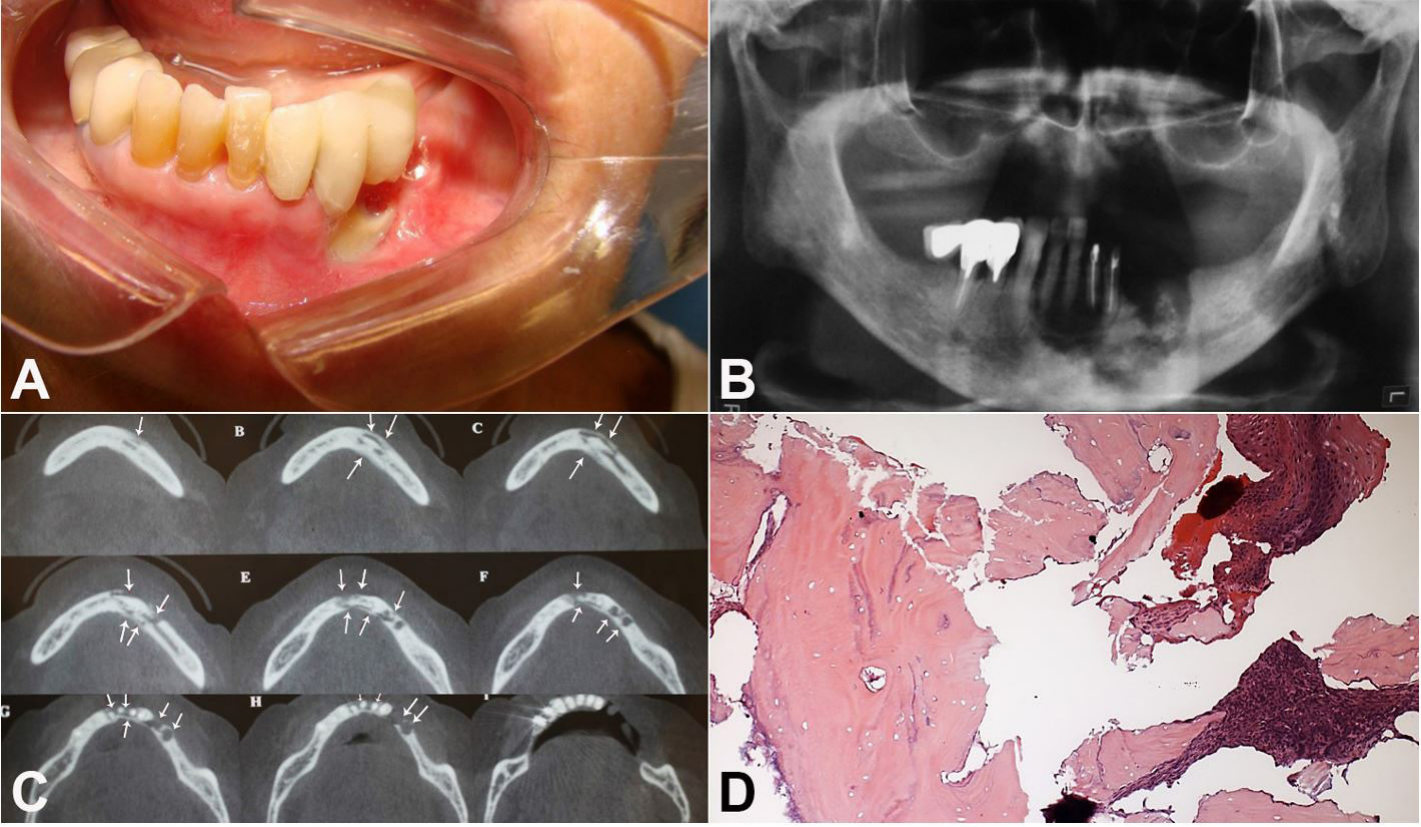
**A** – Exposure of bone and alveolar ridge in the region of teeth #33 and #34 an intraoral aspect; **B** – Area of bone sequestration spreads across the body of the mandible (symphysis region) to the region of pre-molars; **C** – Cone-beam computed tomography; **D** –Histological exam – H&E stained section showing completely necrotic bone trabeculae

Clinical examination showed the exposure of bone and alveolar ridge in the region of teeth #33 and #34 (Figure 1A). Panoramic radiograph demonstrated evidence of radiopaque image suggestive of bone necrosis involving the region of lower incisors. An extensive area of bone sequestration spread across the body of the mandible (symphysis region) to the region of pre-molars (Figure 1B). Cone-beam computed tomography showed a radiolucent, well-defined injury, covering the area of teeth #35 to #43, with impairment of buccal and lingual cortical bones, but with mandibular basal lamina preserved (Figure 1C).

After the incisional biopsy, histological sections stained with hematoxylin/eosin revealed a fragment of lamellar bone tissue with uniform distribution of lacunae and empty feeding canals, including colonies of bacteria closely related to it (Figure 1D).

The patient was diagnosed with osteonecrosis associated with the use of bisphosphonate (sodium ibandronate). She was surgically treated with removal of bone sequestration and teeth (Figures 222C), including antibiotic therapy.

**Figure 2:**
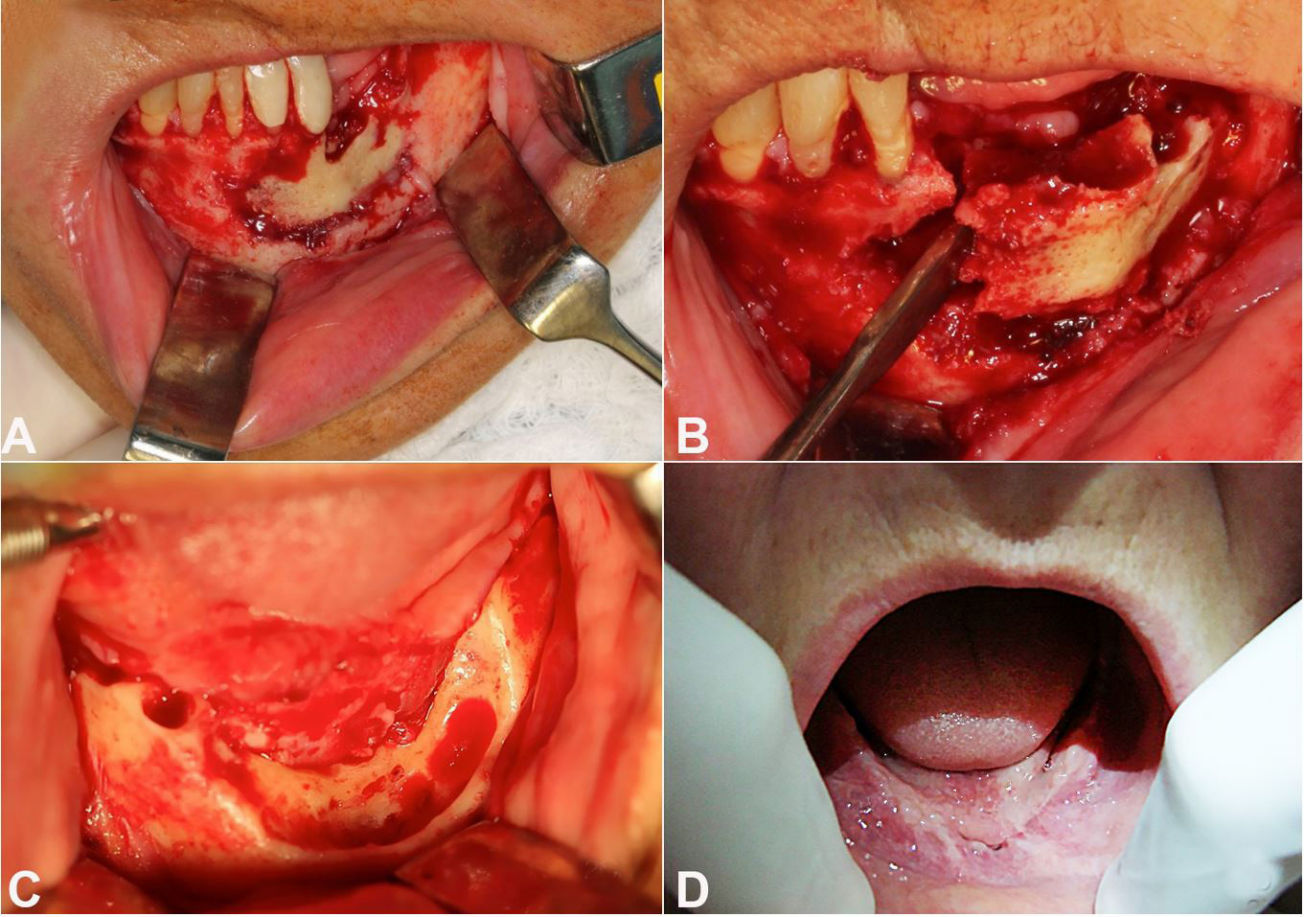
**A** – Necrotic area involving bone and teeth; **B** – Surgical treatment with removal of bone sequestration; **C** – After the removal of bone sequestration and teeth; **D** – Surgical follow up (7 days). No sign of new bone exposure and/or surgical wound dehiscence.

After the surgery, the patient presented proper healing and no sign of new bone exposure and/or surgical wound dehiscence (Figure 2D).

The patient was followed up for six years (a total of 6 appointments), presenting good general health, no sign of bone exposure, and did not have paresthesia. Imaging findings showed no changes related to BRONJ either.

## DISCUSSION

Oral BPs have been usually used for the treatment of osteoporosis. Despite the low incidence of BRONJ in these patients,^7^ some cases have already been reported^8^ ^,^ ^9^ ^,^ ^11^ ^,^ ^12^ and presented high morbidity, leading to pathological fractures.^12^

The patient, who develops osteoporosis, belongs, almost always to the age group with other systemic chronic diseases, which are also additional risk factors for BRONJ. Diseases like diabetes, hypertension, dyslipidemia, and rheumatoid arthritis can affect micro-vascularization, and as such they are risk factors for the development of BRONJ.^13^ ^,^ ^14^

In general, the most used drugs in the treatment of osteoporosis are alendronate, risedronate, and ibandronate. These medications can be ordered according to their potency (i.e. risedronate > ibandronate > alendronate) and bone-binding affinity (i.e. alendronate > ibandronate > risedronate).^3^

In the present case, we have identified several risk factors for the development of BRONJ, namely: age, comorbidities potentially affecting vascularization, and the use of ibandronate. Although the literature suggests that the risk of BRONJ development in patients on oral BPs might be associated with longer use of them (i.e., > 3 years), this observation is particularly related to the use of alendronate.^15^ ^-^ ^17^ Ibandronate is the least reported drug in epidemiological surveys, but case reports are pointing to the development of BRONJ after 2 years of continuous use of the drug.^8^ ^,^ ^9^ ^,^ ^11^ ^,^ ^12^

Another important issue is the choice of treatment of BRONJ, which had been extremely conservative, based on antibiotics and letting invasive measures for extensive lesions.^14^ The literature has recently shown that the disease can progress if not treated or conservatively controlled. Early intervention with surgical resection should be the preferred method of treating any disease stage, resulting in better outcomes and decreasing its morbidity.^18^ This information has encouraged us to carry out direct, conclusive treatment, and therefore we opted for a surgical approach regarding the affected bone. Although the treatment has been effective, the delay in performing it led to a substantial loss of hard tissue, which did not allow us to achieve the patient’s oral rehabilitation.

Prior to the surgical procedure, the patient’s doctor discontinued the use of ibandronate. This is still a point of discussion. The American Association of Oral and Maxillofacial Surgeons, in its position paper, recommends (on the empirical basis) the discontinuation of oral BPs three months before and three months after the surgery. However, multicentric prospective studies showed that such recommendation has no benefit regarding the prevention of BRONJ, regardless of the administration route (i.e., oral or intravenous).^6^ ^,^ ^19^

In addition, a BP drug holiday can increase the risk of osteoporotic femur fracture.^20^ The significant mortality rate in patients after osteoporotic fractures^21^ and the low incidence^7^ and high likelihood of complete BRONJ healing^18^ lead us to recommend that BPs should not be discontinued in osteoporotic patients.
